# NOTCH4 Exhibits Anti-Inflammatory Activity in Activated Macrophages by Interfering With Interferon-γ and TLR4 Signaling

**DOI:** 10.3389/fimmu.2021.734966

**Published:** 2021-12-01

**Authors:** Susana López-López, María José Romero de Ávila, Natalia Carolina Hernández de León, Francisco Ruiz-Marcos, Victoriano Baladrón, María Luisa Nueda, Jorge Laborda, José Javier García-Ramírez, Eva M. Monsalve, María José M. Díaz-Guerra

**Affiliations:** ^1^ Medical School, Centro Regional Investigaciones Biomedicas (CRIB)/Biomedicine Unit, University of Castilla-La Mancha/Centro Superior Investigaciones Científicas (CSIC), Albacete, Spain; ^2^ University Hospital Complex of Albacete, Albacete, Spain; ^3^ Pharmacy School, Centro Regional Investigaciones Biomedicas (CRIB)/Biomedicine Unit, University of Castilla-La Mancha/Centro Superior Investigaciones Científicas (CSIC), Albacete, Spain

**Keywords:** macrophage, inflammation, NOTCH4, IFN-γ, TLR

## Abstract

NOTCH4 is a member of the NOTCH family of receptors whose expression is intensively induced in macrophages after their activation by Toll-like receptors (TLR) and/or interferon-γ (IFN-γ). In this work, we show that this receptor acts as a negative regulator of macrophage activation by diminishing the expression of proinflammatory cytokines, such as IL-6 and IL-12, and costimulatory proteins, such as CD80 and CD86. We have observed that NOTCH4 inhibits IFN-γ signaling by interfering with STAT1-dependent transcription. Our results show that NOTCH4 reprograms the macrophage response to IFN-γ by favoring STAT3 *versus* STAT1 phosphorylation without affecting their expression levels. This lower activation of STAT1 results in diminished transcriptional activity and expression of STAT1-dependent genes, including IRF1, SOCS1 and CXCL10. In macrophages, NOTCH4 inhibits the canonical NOTCH signaling pathway induced by LPS; however, it can reverse the inhibition exerted by IFN-γ on NOTCH signaling, favoring the expression of NOTCH-target genes, such as *Hes1*. Indeed, HES1 seems to mediate, at least in part, the enhancement of STAT3 activation by NOTCH4. NOTCH4 also affects TLR signaling by interfering with NF-κB transcriptional activity. This effect could be mediated by the diminished activation of STAT1. These results provide new insights into the mechanisms by which NOTCH, TLR and IFN-γ signal pathways are integrated to modulate macrophage-specific effector functions and reveal NOTCH4 acting as a new regulatory element in the control of macrophage activation that could be used as a target for the treatment of pathologies caused by an excess of inflammation.

## Introduction

Inflammation is a basic defense response induced by the cells of the innate immunity, especially macrophages, against infection and tissue damage. Inflammation allows the recruitment of cells and triggering of effector defense mechanisms to the infectious foci to achieve their elimination. However, deregulation of this process could lead to chronic or excessive inflammation. This produces toxicity and tissue damage that are at the origin of pathologies as severe and diverse as rheumatoid arthritis, atherosclerosis, diabetes or septic shock (1). Macrophage recognition of pathogens by Toll-like receptors (TLRs) is essential for the synthesis and release of proinflammatory cytokines, such as tumor necrosis factor α (TNFα), interleukin 6 (IL-6) or interleukin 12 (IL-12), which organize the development of the inflammatory response and the initiation of an adaptive immune response (2). Signaling through TLRs involves the recruitment of different adaptive proteins that allow the activation of the IκB kinase complex, and mitogen activated protein kinases (MAPKs). This leads to the activation of key transcription factors, such as NF-κB, that increase the expression of pro-inflammatory cytokines (3).

IFN-γ is the mayor product of the adaptative CD4 Th1 cells. This cytokine dramatically stimulates macrophage activation by synergizing with TLRs to induce augmented production of inflammatory cytokines. IFN-γ signaling is mediated by the JAK/STAT pathway. IFN-γ receptor binding leads to the union of JAK1 and JAK2 kinases, which phosphorylate an intracytoplasmic receptor tyrosine residue that serves as a docking site for STAT1 proteins, which are in turn phosphorylated. STAT1:STAT1 homodimers are the main mediators of IFN-γ-dependent transcription, although STAT1-independent pathways involved in the regulation of gene expression by IFN-γ signaling have been suggested (4, 5). IFN-γ also suppresses TLR-induced feedback inhibitory mechanisms, such as those mediated by IL-10 and STAT3 (6, 7), or that mediated by the NOTCH-target genes *Hes1* and *Hey1*, which act as transcriptional repressors (8).

Different studies have shown a relevant role of NOTCH receptors in the control of macrophage activation and the induction of the inflammatory response (9–11). NOTCH receptors are activated by the binding of ligands belonging to of the Delta/Jagged protein families present on adjacent cells. After that, NOTCH receptors are cleaved at the internal side of the membrane and the NOTCH-intracellular domains (NICD) are released and translocated to the nucleus, where they can modulate the expression of different genes. NICD interacts with CBF-1/RBP-J DNA-binding proteins to form a transcriptional activator through the recruitment of coactivator proteins (12). Mammals express four NOTCH genes, NOTCH1-4, which are differentially expressed in the tissues (13). NOTCH4 is widely expressed in blood vessels and plays an important role in the angiogenesis process. However, *Notch4* ^-/-^ animals are viable, even though they show discrete alterations in vascular development (14). The activity of NOTCH4 is controversial, as it appears that this receptor is not easily activated by the ligands, and that its intracellular domain is hardly processed by the mechanism explained above (14). A recent study has shown that *Notch4* ^-/-^ mice are more resistant to mycobacterial infection than controls, due to increased cytokine production. This suggests that NOTCH4 is a negative regulator of the inflammatory response (15), contrary to the effects mediated by NOTCH1 and NOTCH3, which clearly enhance this response (9, 16). NOTCH4 seems to inhibit TLR signaling by affecting TAK1 activation (15). NOTCH4 has been identified also as a protective factor for lung inflammation caused by exposure to ozone (17).

In this study, we show that NOTCH4 inhibits proinflammatory macrophage activation by limiting the production of the cytokines IL-6 and IL12, and the costimulatory proteins CD80 and CD86. We demonstrate for the first time that NOTCH4 activity inhibits IFN-γ signaling by diminishing STAT1 phosphorylation and activation, whereas it enhances STAT3 activation. NOTCH4, through the inhibition of IFN-γ signaling, also favors overall NOTCH signaling by promoting the feedback inhibitor cycle carried out by HES1 and HEY1, which decreases the production of cytokines. These results provide new insights into the mechanisms by which NOTCH, TLR and IFN-γ signals are integrated to modulate specific effector functions in macrophages and reveal NOTCH4 as a new regulatory agent in the control of macrophage activation that could be used as a target for the treatment of pathologies caused by an excessive inflammatory response.

## Materials and Methods

### Mice

All procedures carried out with mice followed the European and Spanish regulations and were approved by the Ethics in Animal Care Committee of the University of Castilla-La Mancha. All mice used in this work were CL57BL/6 wild type (Jackson Laboratories, Farmington, CT, USA).

### Cells and Reagents

Peritoneal macrophages were isolated from three-month-old male mice, four days after i.p. injection of 2 mL 3% sterile thioglycolate broth (w/v in water, Thermo Fisher, Spain) as previously described (18). Elicited macrophages were seeded at 1x10^5^ cells/cm^2^ in complete DMEM medium (supplemented with 10% FBS -Fetal Bovine Serum, 4 mM L-glutamine, and 1% penicillin-streptomycin, all from Lonza, Spain), and incubated overnight in complete DMEM medium supplemented with 2% FBS, preceding activation with either 100 ng/ml LPS from *Salmonella typhimurium* (L7261 SIGMA, Merck Life Science, Spain), and/or 10 U/ml of IFN-γ (Roche, Manheim, Germany). Activation was verified by the Griess test (Griess reagent 215-981-2, Merck Life Science, Spain) (19).

Raw 264.7 cells (ATCC N°. TIB-71) were subcultured at 6–8x10^4^ cells/cm^2^ in DMEM medium (Lonza) supplemented with 10% FBS, 4 mM L-glutamine and 1% penicillin-streptomycin, and incubated overnight in complete DMEM supplemented with 5% FBS, preceding activation with 100 ng/ml LPS from *Salmonella typhimurium*, and/or 10 U/ml IFN-γ. Activation was verified by the Griess test.

Human monocytes were isolated from blood of healthy donors by centrifugation on Ficoll-PaqueTM PLUS (Amersham Biosciences, UK), following standard protocols (20) and cultured in complete DMEM medium supplemented or not with 100 ng/mL LPS for 24 h. Human samples were obtained and processed under the European Union and Spanish regulations.

### Cell Transfections

For transient transfections, 2.5x10^5^ Raw 264.7 cells per well were seeded in triplicate on 24-well plates and transfected on the following day with Lipofectamine 2000 (Thermo Fisher), according to the manufacturer’s recommendations, using OPTI-MEM medium (Thermo Fisher) without supplements and 0.5 µg of total EndoFree plasmid DNA per well. The reporter plasmids pSTAT-luc, pNFκB-luc, pCBF-luc, used to detect STAT1 or STAT3, NFκB and NOTCH-dependent transcription activities, respectively, have been previously described (10, 18) pRLTK Renilla-expressing vector (Promega, Madison, WI, USA) was used as a control for transfection efficiency. pLNCX2 (empty vector), pLNCX2-Hes1 (murine *Hes1* expression vector), pLNCX2-NIC1 (murine active intracellular Notch1), Sh-Control (SA Bioscience, Germantown, MD, USA, 20070705-2), murine Sh-Notch1 (SA Bioscience, KM04747N), Sh-Hes1 (SA Bioscience, KM05647N), Sh-Control (pGeneClip neomycin empty vector, Qiagen, DC24-1), murine Sh-Notch2 (Qiagen, Germantown, MD, USA, 201204125), murine Sh-Notch3 (Qiagen, 201206045), murine Sh-Notch4 (Qiagen, 201204121), Sh-Control (pLKO.1 Sigma-Aldrich), Sh-STAT3 (Sigma, TRCN0000071456), pCMV6 entry (empty vector), pCMV6 entry-Notch3 (murine *Notch3* expression vector), pcDNA (empty vector), pcDNA-Notch2 (murine *Notch2* expression vector), PCD2 (empty vector), pEGFN1 (empty vector), PEGFN1-Notch4 (murine *Notch4* expression vector) and/or PCD2-Notch1 (full-length murine *Notch1* expression vector) (21) were used together with the reporters.

Cells were stimulated with 100 ng/mL LPS and/or 10 U/ml of IFN-γ 24 h after being transfected for the indicated times. Luciferase and Renilla activities were measured by using the Dual Luciferase Reporter Assay System (Promega) in a Sirius luminometer (Berthold) following the manufacturer’s recommendations.

For transient transfections to obtain RNA and protein, 1.5x10^6^ cells were seeded on 35 mm plates 24 h before transfection and transfected on the following day with Lipofectamine 2000 (Invitrogen) and 4 µg per plate of EndoFree plasmid DNA of PEGFN1, PEGFN1-N4, Sh-Control or Sh-Notch4, using OPTI-MEM medium (Gibco). 24 hours after transfection cells were activated with LPS and IFN-γ at different times. Supernatants were recovered and assayed for IL-6 by ELISA, using the Mouse IL-6 ELISA Ready-Set-Go kit from eBioscience.

To limit STAT3 activation, inhibitors for each of the enzymes responsible for its activation were used: for JAK2, Jak Inhibitor I 10 µM (Santa Cruz 204021); for SCR, PP1 10 µM (Abcam, UK, 120859). To inhibit protein synthesis, 1 µg/ml cycloheximide (CHX) was used (Sigma-Aldrich) for the indicated times.

### Protein Extracts and Western Blot Analysis

Cells were washed twice with ice-cold PBS, scraped off the dishes and collected by centrifugation. Cell pellets were resuspended in RIPA lysis buffer (25 mM Hepes, pH 7.5; 1.5 mM MgCl_2_; 0.2 mM EDTA; 1% Triton X-100; 0.3 M NaCl; 20 mM β-Glycerophosphate; 0.1% SDS; 0.5% desoxycholic acid) supplemented with a cocktail of protease inhibitors and I and II phosphatase inhibitors (Sigma-Aldrich), homogenized for 30 min. at 4°C and centrifuged at 8,000 x g for 15 min. Protein concentrations were determined by the bicinchoninic acid method (Bio-Rad, Hercules, CA, USA).

Denatured total protein extracts (40-80 µg) were separated by SDS-PAGE in 10% polyacrylamide gels, transferred to PVDF membranes (Sigma-Aldrich) and processed according to the antibody suppliers’ recommendations. Proteins were detected with ECL (Santa Cruz, Biotechnology Dallas, TX, USA). β-tubulin or ERK expression were used as loading controls. When possible, different antibodies were used consecutively in the blots; if the expected molecular weight of the proteins was similar, then the bound antibodies were removed by incubation with a stripping buffer (100mM B-mercaptoethanol, 2% Sodium dodecyl sulfate, 62,5 mM Tris HCl pH 6.7) at 55°C for 30 minutes prior to the second blotting.

Anti-IRF1 (D5E4), anti-pSTAT1 (Tyr701) (58D6), anti-Pstat3 (Tyr705) (D3A7), anti-JAK2 (D2E12) and anti-STAT1 (9172S) were purchased from Cell Signaling (Danvers, MA, USA). Anti-NOTCH1 (C-20), anti-ERK (sc-154), and anti-STAT3 (F-2) from Santa Cruz; Anti-ADAM10 (ab19026) and anti-NOTCH 4 (07-189) were purchased from Merck; anti-Furin (ab3467) and anti-NOTCH 4 (ab184742) from Abcam; and anti-α-tubulin from Sigma-Aldrich.

### RNA Purification and cDNA Synthesis

Total RNA was obtained by using the RNeasy Kit (Qiagen) with DNase (Promega), according to the manufacturer’s instructions, and quantified in a ND-1000 (NanoDrop) spectrophotometer. cDNA was synthesized from 1 µg of total RNA by using the RevertAidH Minus First Strand cDNA Synthesis Thermo Scientific, following manufacturer´s recommendations.

### Quantitative RT-PCR

Gene expression analysis by quantitative RT-PCR (qRT-PCR) was performed in triplicate according to the Fast SYBR^®^Green Protocol with the StepOne real-time PCR detection system (Applied Biosystems). Specific oligonucleotides were designed with the PrimerQuest SM computer program (Integrated DNA technologies, Inc.Coralville IA, USA) and are indicated in **Table 1**. The mRNA levels of mouse riboprotein P0 (22) or human GAPDH were used as internal controls.

**Table 1:** Oligonucleotides used for PCR.

Gene	Forward Primer	Reverse Primer
mP0	5′-GAATCGCTCCTGCAGCAAAG-3′	5′-CCAGGGTCTCATCCGCATT-3′
mHes1	5′-AAGCGCGTCCTGGCATTGTCT-3′	5′-CCGCAGGGGCAGCAGTGGT-3′
mFurina	5′-ACACACAGATGAATGACAAC-3′	5′-GCATTGTAA GCT ACA CCT AC-3′
mNotch1	5′-TGTCTATGCCAGGCTAATGAAG-3’	5’-AGGGTGAGCAGGAACATGAG-3’.
mNotch4	5′-TGAATCGGAGGTTCTGGATGTGGA-3’	5′-AGTGGTTCCCAGGGTTCCAGATTT-3’
hNotch4	5’-ACACACACATGAGGATCTCTGGCA-3’	5’-AGTTGGCCTTGTCTTTCTGGTCCT-3’
hGADPH	5’-AACCCTTGGCATTGTGGAAGG-3’	5’-GGATGCAGGGATGATGTTCT-3’
mIrf1	5′-GAATCGCTCCTGCAGCAAAG-3’	5′-CCAGGGTCTCATCCGCATT-3’
P40	5′-GCACGGCAGCAGAATAAATATG-3’	5′-GGTTTGATGATGTCCCTGATGA-3’
IL6	5′-CCACGGCCTTCCCTACTTC-3’	5′-TTGGGAGTGGTATCCTCTGTGA-3’
mJAK1	5-’GATGAGAGAAACAAACTCCG-3’	5’-GCTTGAGTTCCATGTTTTTG-3’
mJAK2	5′-CTTATAAACCTGGAAACCCTG-3’	5′-TAACTGTACGTCCTGTTCTG-3’
mFgr	5’-AAGAGTGGTACTTCGGAAG-3’	5′-TAATGCTTTATGTGATCGCC-3’
mHck	5’-CTACATCCCAAGCAACTATG-3’	5’-CAAAGTCTCGAACAGACAAC-3’
mLyn	5’-GAAGCCATGGGATAAAGATG-3’	5’-TGTTATAGTAACCCATCCAGAC-3’
mIfngr1	5’-CCTGTTACACATTCGACTATAC-3’	5’-TTGCCAGAAAGATGAGATTC-3’
mStat1	5’-AGGAAAATCAAGACCCTAGAAG-3’	5’-CTCCTTTCTCTTATTGTCAAGC-3’
mStat3	5’-CGTCTGGAAAACTGGATAAC -3’	5’-TTAAGTTTCTGAACAGCTCC-3’
mSOCS1	5’-CTGAATTCCACTCCTACCTC-3’	5’-AGAAAAATGAAGCCAGAGA-3’
mSOCS3	5’ATTGGCTGTGTTTGGCTCCTTGTG-3’	5’-AGCAGATGGAGGGTTCTGCTTTGT3’
mCXCL10	5’-CCTTGGGAAGATGTGGTTAAG-3’	5’-TCAGGCTCGTCAGTTCTAAGT-3’
mATF3	5’-TCAAGGAAGAGCTGAGATTCGCCA-3’	5’-GTTTCGACACTTGGCAGCAGCAAT3’
mIL10	5’-CCTGGATCTGTATCACCGAAGC-3’	5’-CTCCGACCACTCTGCCTTGTTA-3’

### Statistical Analysis

The student’s unpaired *t*-test was used for statistical analyses between two groups, and one-way ANOVA/Bonferroni’s post-tests were performed for statistical analyses of more than two groups.

## Results

### NOTCH4 Expression Is Induced During Macrophage Activation

**Figure 1:**
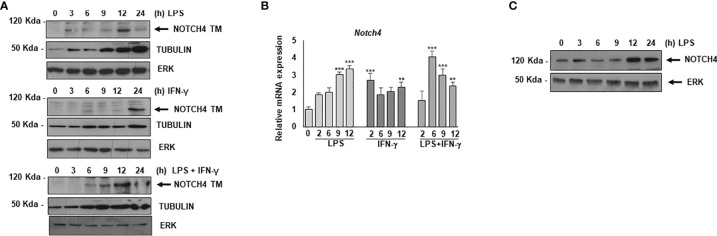
Analysis of NOTCH4 expression after macrophage activation by TLR4 agonists and IFN-γ. **(A)** Western blot analysis for NOTCH4 expression in peritoneal macrophages activated with LPS (100 ng/ml) and/or IFN-γ (10 U/ml) for different times. β-tubulin expression was used as a loading reference. The image is representative of three independent experiments. TM mature transmembrane receptors **(B)** qRT-PCR analysis of *Notch4* gene expression in peritoneal murine macrophages activated with LPS in the presence or in the absence of IFN-γ for different times. Means ± SD of three independent experiments are shown. One-way ANOVA/Bonferroni’s post-tests were performed. Statistical significance was determined at the level of **p<0.01, ***p<0.001. **(C)** Western blot analysis of NOTCH4 expression in human monocytes activated with LPS for different times. The image is representative of three independent experiments. ERK expression was used as a loading reference.

We first analyzed the expression of NOTCH4 by Western blot in TLR4, and/or IFN-γ- activated macrophages (**Figure 1A**). We observed that, as previously described for NOTCH1 and NOTCH2, NOTCH4 was not expressed in control differentiated macrophages (9); nevertheless, its expression increased in all cases about six hours after macrophage activation and remained elevated for at least 24 h (**Figure 1A**). Accordingly, *Notch4* mRNA levels were increased after macrophage activation (**Figure 1B**).

NOTCH4 expression was also detected in human monocytes. In this case, basal levels were observed in control, non-activated cells, but higher NOTCH4 expression was detected in LPS-activated monocytes 12-24h after activation (**Figure 1C**).

### NOTCH4 Inhibits the Expression of Some Proinflammatory Cytokines and Costimulatory Proteins in Macrophages

**Figure 2:**
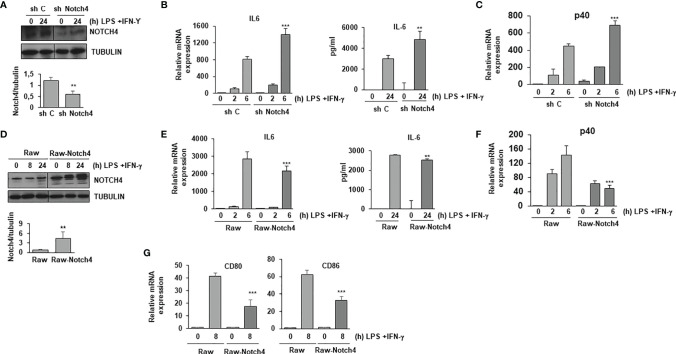
NOTCH4 inhibits the expression of pro-inflammatory cytokines in TLR4 and IFN-γ activated macrophages. **(A)** Western blot analysis of NOTCH4 expression in Raw 264.7 cells transiently transfected with control scrambled shRNA (sh C) or *Notch4* shRNAs. One day after transfection, cells were stimulated with LPS (100 ng/ml) and IFN-γ (10 U/ml) for 24 h before analysis. A representative experiment is shown. Quantitation of NOTCH4 protein levels 24h after transfection is shown. Means ± SD of at least three independent transfections are shown ** indicates statistical significance (p<0.01) respect to control conditions **(B)** qRT-PCR (left panel) and ELISA (right panel) analysis of IL-6 expression in Raw 264.7 cells transiently transfected with control or *Notch4* shRNAs for 24 hours and activated with LPS and IFN-γ for the indicated times. Means ± SD of at least three independent experiments are shown. One-way ANOVA/Bonferroni’s post-tests were performed, **p<0.01 and ***p<0.001, compared to the corresponding treatment of the scrambled shRNA. **(C)** qRT-PCR analysis of p40 IL-12 expression in Raw 264.7 cells activated as described above. Means ± SD of at least three independent experiments are shown. One-way ANOVA/Bonferroni’s post-tests were performed, ***p<0.001 compared to the corresponding treatment of the scrambled shRNA. **(D)** Western blot analysis of NOTCH4 expression in Raw 264.7 cells transiently transfected with a *Notch4* expression vector (Raw-Notch4) or with the corresponding control empty vector (Raw). One day after transfection, cells were stimulated with LPS and IFN-γ for different times before analysis. A representative experiment is shown. Quantitation of NOTCH4 protein levels 24h after transfection is shown. Means ± SD of at least three independent transfections are shown **(E)** qRT-PCR (left panel) and ELISA (right panel) analysis of IL-6 expression in Raw 264.7 cells transiently transfected with a *Notch4* expression vector (Raw-Notch4) or with the corresponding empty vector (Raw) for 24 hours and activated with LPS and IFN-γ for the indicated times. Means ± SD of at least three independent experiments are shown. One-way ANOVA/Bonferroni’s post-tests were performed, **p<0.01 and ***p<0.001, compared to the corresponding treatment of the empty vector transfected cells. **(F)** qRT-PCR analysis of p40 IL-12 expression in Raw 264.7 cells activated as described above. Means ± SD of at least three independent experiments are shown. One-way ANOVA/Bonferroni’s post-tests were performed, ***p<0.001 compared to the corresponding treatment of the empty vector transfected cells. **(G)** qRT-PCR analysis of CD80 and CD86 expression in Raw 264.7 cells activated for 8 hours with LPS and IFN-γ. Means ± SD of at least three independent experiments are shown. One-way ANOVA/Bonferroni’s post-tests were performed, ***p<0.001 compared to the corresponding treatment of the empty vector transfected cells.

A previous report has shown that mice lacking the NOTCH4 receptor are more resistant to infection by *Mycobacterium tuberculosis* (15). This suggests that the presence of NOTCH4 reduces in some way the effectiveness of the immune system in the defense against this pathogen. To study the reason for this, we analyzed the effect of NOTCH4 activity in macrophage activation by evaluating the expression of some proinflammatory cytokines. As **Figure 2** shows, Raw 264.7 cells forced to express reduced NOTCH4 levels with a specific shRNA treatment (**Figure 2A**) presented higher expression of IL-6 (**Figure 2B**) or p40 IL-12 (**Figure 2C**) than control cells. Accordingly, cells overexpressing NOTCH4 (**Figure 2D**) presented lower expression of these cytokines after activation with LPS and INF-γ (**Figures 2E, F**), arguing for a role of NOTCH4 in the control of proinflammatory cytokines expression.

We also evaluated the expression levels of the costimulatory proteins CD80 and CD86 in macrophages, as these proteins are essential for T cell activation. As **Figure 2G** shows, higher levels of NOTCH4 expression reduced the expression of these two proteins in macrophages after activation with LPS and IFN-γ, suggesting a role of NOTCH4 in the capacity of macrophages to fully activate T cells.

### NOTCH4 Inhibits the Response of Macrophages to IFN-γ

**Figure 3:**
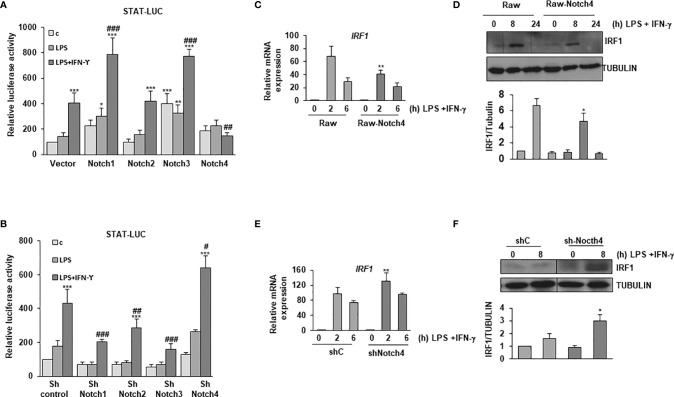
Effect of NOTCH4 expression on the response of LPS-activated macrophages to IFN-γ. Analysis of the luciferase activity in Raw 264.7 cells transiently transfected with a STAT-luciferase reporter (STAT-luc) and **(A)** different *Notch* receptor expression vectors or the corresponding empty vector or **(B)** with specific shRNAs for the different *Notch* receptor genes or control shRNA. One day after transfection, cells were stimulated with LPS (100 ng/ml) and IFN-γ (10 U/ml) for 24 h before analysis. The means ± SD of three independent experiments are shown. One-way ANOVA/Bonferroni’s post-tests were performed. *p<0.05, **p<0.01 and ***p<0.001, compared to the control transfected cells. ^#^p<0.05, ^##^p<0.01, ^###^p<0.001, compared to LPS and IFN-γ treated control transfected cells. **(C)** Quantitative PCR and **(D)** Western blot analysis of IRF1 expression in Raw 264.7 cells transfected with a *Notch4* expression vector or the corresponding empty vector for 24 hours and activated for different times with LPS and IFN-γ. RNA expression was referred to that of the *P0* housekeeping gene used as a control. Protein levels were referred to the tubulin loading control. Quantitation of three different experiments is shown. One-way ANOVA/Bonferroni’s post-tests were performed. *p<0.05, **p<0.01, compared to the corresponding treatment of the empty vector transfected cells. **(E)** Quantitative PCR and **(F)** Western blot analysis of IRF1 expression in Raw 264.7 cells transfected with specific shRNA for *Notch4* gene or control shRNA for 24 hours and activated for different times with LPS and IFN-γ. RNA expression was referred to that of the *P0* gene. Protein levels were referred to tubulin loading control. Quantitation of three different experiments is shown. One-way ANOVA/Bonferroni’s post-tests were performed. *p<0.05, **p<0.01, compared to the corresponding treatment of the scrambled shRNA.

IFN-γ is essential for the defense against mycobacteria. In macrophages, the expression of IL-6, IL-12 and the costimulatory proteins CD80 and CD86 is enhanced by IFN-γ. To unveil the potential role of NOTCH4 on IFN-γ signaling, we explored the effect of overexpressing NOTCH4 on the activity of a STAT-dependent reporter gene in macrophages activated by LPS or by LPS and IFN-γ and compared it with the effects caused by overexpressing the other NOTCH receptors. As previously described (10), we observed that elevated NOTCH1 or NOTCH3 expression levels increased STAT-dependent reporter activity, whereas NOTCH2 had no significant effect. Interestingly, when NOTCH4 was overexpressed, a dramatic inhibition of the STAT-dependent reporter activity was observed (**Figure 3A**). We then used specific shRNAs to selectively decrease the expression of each NOTCH receptor, and, accordingly with the results described above, we observed that diminished expression of NOTCH1 and NOTCH3 lead to a decrease in the STAT-dependent reporter activity, whereas an increase in this activity was observed when decreasing NOTCH4 expression in Raw 264.7 cells activated with LPS and IFN-γ (**Figure 3B**). Similar changes in the STAT-dependent reporter activity were observed when Raw 264.7 cells were activated only with IFN-γ and NOTCH4 expression was altered (**Supplementary Figure 1**). All our data showed that NOTCH4 exerts a negative regulation on IFN-γ signaling, by limiting STAT-dependent transcription.

IRF-1 is a transcription factor involved in the signaling cascades triggered by type I and II interferons, and its promoter contains gamma-activated sequence (GAS) sites that bind STAT1 (23). To confirm previous results, we determined the IRF-1 expression levels in macrophages with modified NOTCH4 expression. As shown in **Figures 3C, D**, treatment of Raw 264.7 cells with LPS and IFN-γ caused an increase in IRF-1 expression, both at the mRNA and protein levels; however, that induction was lower in *Notch4*-transfected cells. On the contrary, the decrease in *Notch4* expression by shRNAs caused a more intense induction of IRF-1 after treatment with LPS and IFN-γ (**Figures 3E, F**). Similar NOTCH4-dependent effects were observed when the expression of other STAT1-dependent genes, such as SOCS1 or CXCL10 was analyzed after activation with LPS and IFN-γ or with IFN-γ alone (**Supplementary Figure 2**).

**Figure 4:**
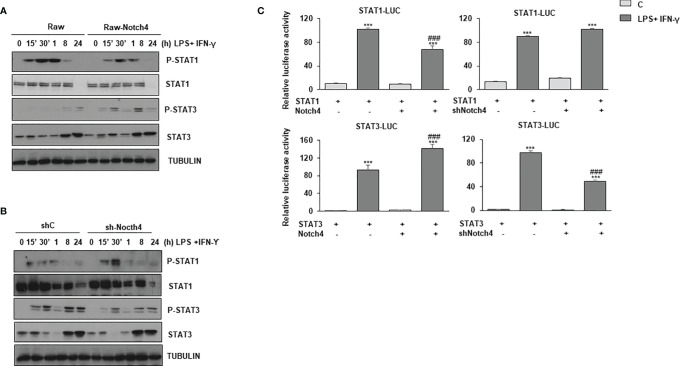
Effect of NOTCH4 expression on the phosphorylation and activation of STAT1 and STAT3 in macrophages activated with IFN-γ and LPS. Analysis by Western blot of STAT1 and STAT3 tyrosine phosphorylation in Raw 264.7 cells transiently transfected with a *Notch4* expression vector or the corresponding empty vector **(A)**, or with specific shRNA for *Notch4* gene or control shRNA **(B)**. One day after transfection, cells were stimulated with LPS (100 ng/ml) and IFN-γ (10 U/ml) for different times before analysis. A representative experiment out of three is shown. **(C)** Analysis of luciferase activity in Raw 264.7 cells transiently transfected with a STAT-luciferase reporter in the presence of STAT1 (STAT1-LUC) (upper panels) or STAT3 (STAT3-LUC) (Lower panel) and a *Notch4* expression vector or the corresponding empty vector (right panels) or with specific shRNA for *Notch4* or control shRNA (lefth panels). One day after transfection, cells were stimulated with LPS (100 ng/ml) and IFN-γ (10 U/ml) for 24 h before analysis. The means ± SD of three independent experiments are shown. One-way ANOVA/Bonferroni’s post-tests were performed. ***p<0.001 compared to the control transfected cells. ^###^p<0.001 compared to LPS and IFN-γ treated control transfected cells.

STAT1 and, to a lesser extent, STAT3, are rapidly phosphorylated in specific tyrosine residues after LPS and IFN-γ activation. We next analyzed STAT1 Tyr701 phosphorylation and STAT3 Tyr705 phosphorylation both in control Raw 264.7 cells (Raw-vector) and in Raw 264.7 cells stably transfected with a *Notch4* expression vector (Raw-Notch4), activated or not with LPS and IFN-γ. As shown in **Figure 4A**, Raw-Notch4 cells presented lower phosphorylation of STAT1 than control cells; however, NOTCH4 did not affect the expression levels of STAT1 protein, which were similar in both cell types. We also observed that the expression of STAT1 diminished 24 hours after activation with LPS and IFN-γ in both cell types, probably due to ubiquitination and proteosome degradation, previously described as a negative feedback regulatory mechanism of IFN-γ signaling (24, 25). On the contrary, we observed higher STAT3 Tyr705 phosphorylation in Raw-Notch4 cells, although STAT3 expression levels were not altered. To validate these results, we analyzed the phosphorylation levels of these proteins after decreasing *Notch4* expression by specific shRNAs. Raw 264.7 cells stably transfected with a *Notch4* shRNA showed enhanced STAT1 phosphorylation when cells were activated with LPS and IFN-γ, whereas in the same conditions, STAT3 phosphorylation level decreased (**Figure 4B**). STAT3 phosphorylation presents a bi-phasic activation, a rapid phosphorylation (15-30 min) mediated by IFNγ receptor activity, that is potentiated by NOTCH4, and a late phosphorylation (8-24h) probably consequence of the paracrine effect of IL-10, secreted after activation with Toll4 ligand or IFNγ and potentiated by NOTCH4 (**Figures 4A, B**). pSTAT3 can be polyubiquitinated and degraded in the proteasome after activation, thereby disrupting STAT3-mediated gene activation (26). This could probably be the reason why STAT3 protein disappears at 30min-1h after activation. Similar effects of NOTCH4 in STAT1/3 phosphorylation were observed in Raw 264.7cells activated only with IFNγ (**Supplementary Figure 3**).

We next analyzed the effect of NOTCH4 on STAT1 and STAT3 transactivation, by using a STAT-dependent reporter gene and expression vectors for STAT1 or STAT3, in cells transfected with a *Notch4* expression vector or treated with *Notch4* shRNAs. As **Figure 4C** shows, in activated macrophages, high levels of NOTCH4 expression diminished STAT1 transcriptional activity, whereas that of STAT3 was significantly increased. On the contrary, when the expression of NOTCH4 was diminished by shRNA, STAT1 transactivation activity was slightly increased, whereas that of STAT3 was lowered. To confirm the higher STAT3 activation after LPS and IFN-γ in the presence of NOTCH4, we next evaluated different genes, whose expression depends on STAT3 activity (27), such as ATF3, SOCS3 (**Supplementary Figure 4**). In both cases, our results showed increased gene expression in the presence of higher levels of *Notch4*, as shown also for IL-10 (**Supplementary Figure 4C**), and decreased gene expression when *Notch4* levels were lowered. Similar effects were observed when Raw 264.7cells were activated only with IFNγ (**Supplementary Figure 4D**). These results confirm that NOTCH4 participates in a cross-regulatory mechanism between STAT1 and STAT3 in LPS and IFN-γ activated macrophages.

To try to identify the mechanism by which NOTCH4 inhibits STAT1 phosphorylation and activation, while increasing that of STAT3, after macrophage activation, we analyzed the expression of the α and β IFN-γ receptor subunits, and the expression of JAK1 and JAK2 in Raw 264.7 cells with increased expression of *Notch4*. As shown in **Supplementary Figures 5A, B**, no significant variations were detected. This suggests that the inhibition that NOTCH4 exerted on the IFN-γ signaling is not mediated by a change in the expression of the IFN-γ receptor subunits or their associated kinases, and another mechanism must be responsible for the observed variations in STAT1 and STAT3 transcriptional activities.

Although JAK2 is the main kinase responsible for STAT1 and STAT3 phosphorylation, other kinases have been also implicated in STAT3 phosphorylation, such as some members of the Src family of kinases (28). Although several members of the Src kinase family, such as Fgr, Hck or Lyn, are expressed in macrophages (29) and are induced by LPS and IFN-γ, no changes in their expression levels were appreciated when *Notch4* expression was increased (**Supplementary Figure 5C**). We next used specific JAK2 or Src kinase inhibitors in macrophages activated with LPS and IFN-γ and analyzed STAT1 and STAT3 phosphorylation. When JAK2 was inhibited, we observed that phosphorylation of both STAT1 and STAT3 was completely abolished, either in the presence or in the absence of NOTCH4 (**Supplementary Figure 6**). In addition, a decrease in the amount of STAT1 protein level was observed when JAK2 is inhibited, something expected since *Stat1* is an IFN-γ inducible gene dependent on STAT1 phosphorylation and activation (30). The treatment with the Src inhibitor, as previously described, did not affect STAT1 phosphorylation but, interestingly, it partially diminished the phosphorylation levels of STAT3 (**Supplementary Figure 6**), and, furthermore, a NOTCH4-dependent enhancement of STAT3 phosphorylation was observed. Together, our data suggest that the modulation of JAK2 activity could mediate the observed cross-regulation between STAT1 and STAT3 phosphorylation induced by NOTCH4 in activated macrophages, although we cannot discard that NOTCH4 could act downstream of JAK2.

### NOTCH4 Inhibits NOTCH-Dependent CBF-1 Transcriptional Activity in LPS-Activated Macrophages, But Reverses the Inhibition Exerted by IFN-γ on Global NOTCH Activity

**Figure 5:**
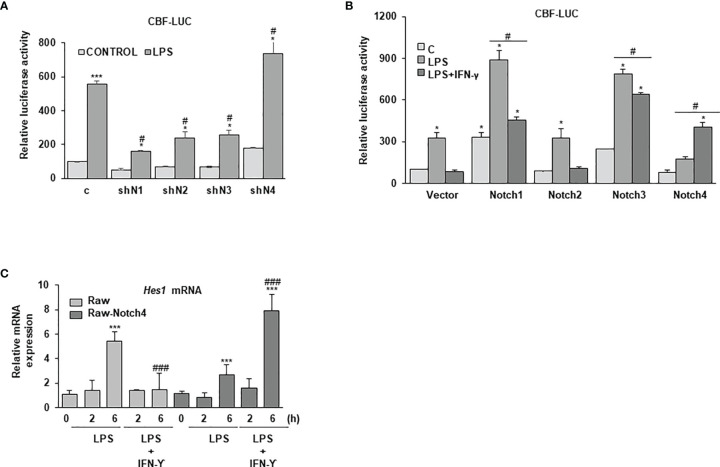
Effect of NOTCH4 on NOTCH transcriptional activity in macrophages activated with LPS and IFN-γ. **(A)** NOTCH transcriptional activity analysis in Raw264.7 cells transiently transfected with a CBF-1 luciferase reporter gene (CBF-luc) and specific shRNAs for the different Notch receptor genes or control shRNAs or **(B)** expression vectors for the different *Notch* receptors or their corresponding empty vectors. One day after transfection, cells were stimulated with LPS (100 ng/ml) **(A)** and/or LPS and IFN-γ (10 U/ml) **(B)** for 24 h before analysis. The means ± SD of three independent experiments are shown. One-way ANOVA/Bonferroni’s post-tests were performed. *p<0.05 compared to the control transfected cells. ^#^p<0.05 compared to the corresponding LPS or LPS and IFN-γ treated control transfected cells. **(C)** Quantitative PCR analysis of *Hes1* expression in Raw 264.7 cells transfected with a *Notch4* expression vector (Raw-Notch4) or its corresponding empty vector (Raw) for 24 hours; cells were then stimulated for different times with LPS or LPS and IFN-γ. RNA expression was referred to that of the *P0* housekeeping gene used as a control. Quantitation of three different experiments is shown. One-way ANOVA/Bonferroni’s post-tests were performed. ***p<0.001 compared to the corresponding control untreated cells. ^###^p<0.001 compared to the corresponding LPS treated cells.

NOTCH4 seems to act in the opposite way to the rest of NOTCH receptors during macrophage activation. Therefore, we decided to evaluate its capacity to signal through the canonical NOTCH pathway by determining the CBF-1-dependent activation of gene transcription. As shown in **Figure 5A**, treatment of macrophages with LPS activated the canonical NOTCH pathway, as reflected by the increase in the activity of a specific luciferase reporter gene under the control of the CBF-1 promoter. This activation was blocked when the expression of NOTCH receptors was diminished with shRNAs, except for NOTCH4, in which case an increase in the CBF-1-activity was observed (**Figure 5A**). Accordingly, NOTCH4 overexpression diminished the activity of CBF-1, whereas overexpression of any of the other three NOTCH receptors increased it (**Figure 5B**). Our results seem to indicate that NOTCH4 does not directly activate CBF-1 as the rest of NOTCH receptors do, but on the contrary, it acts as an inhibitor of the activity of this transcription factor.

Previous reports have clearly demonstrated that IFN-γ inhibits NOTCH signaling in macrophages (8, 9). Our data confirmed that, as shown in **Figure 5B**, treatment with LPS and IFN-γ decreased the CBF-1 reporter activity induced by treatment with LPS alone. CBF-1 inhibition by IFN-γ is also observed when the expression of the different NOTCH receptors is increased, except in the case of NOTCH4. In this instance, CBF-1 dependent transcriptional activity is increased with IFN-γ treatment. One could speculate that the inhibition of IFN-γ signaling exerted by NOTCH4 (**Figure 3**) could be responsible of reversing the effect that IFN-γ exerts on the NOTCH signaling pathway. We observed similar results when we analyzed the expression of the NOTCH-target gene *Hes1*. As **Figure 5C** shows, treatment of Raw 264.7 cells with IFN-γ diminished *Hes1* mRNA expression induced by LPS. However, IFN-γ treatment increased *Hes1* expression induced by LPS in cells expressing high levels of NOTCH4. Our data suggest that NOTCH4 reverses the inhibition that IFN-γ exerts on overall NOTCH activity, allowing IFN-γ to mediate the increase in HES1 expression levels.

**Figure 6:**
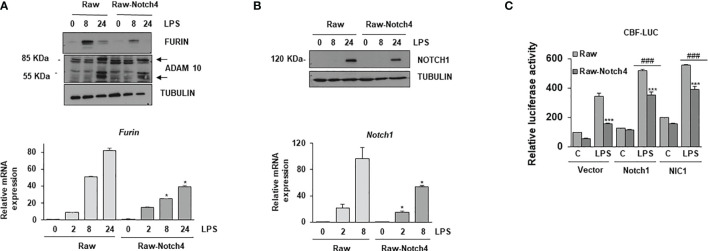
Effect of NOTCH4 expression on NOTCH receptor processing and signaling. **(A)** (Upper panel) Western blot analysis of furin and ADAM10 expression; (lower panel) furin mRNA analysis by RT-PCR in Raw 264.7 cells transiently transfected with a *Notch4* expression vector or the corresponding empty vector, activated with LPS (100 ng/ml) and/or IFN-γ (10 U/ml) for different times. β-tubulin expression was used as a loading reference in Western blots. The image is representative of three independent experiments. RNA expression was referred to that of the *P0* housekeeping genes used as a control. Quantitation of three different experiments is shown. One-way ANOVA/Bonferroni’s post-tests were performed. *p<0.05 compared to the corresponding control treated cells. **(B)** Analysis by Western blot (upper panel) and qRT-PCR (lower panel) of *Notch1* gene expression in Raw 264.7 cells transiently transfected with a *Notch4* expression vector or the corresponding empty vector, activated with LPS and IFN-γ for different times. β-tubulin expression was used as a loading reference in Western blot. The image is representative of three independent experiments. RNA expression was referred to that of the P0 gene. Quantitation of three different experiments is shown. One-way ANOVA/Bonferroni’s post-tests were performed. *p<0.05 compared to the corresponding control treated cells. **(C)** Effect of NOTCH4 expression on NOTCH1 transcriptional activity. Raw 264.7 cells were transfected with a CBF-1 luciferase reporter plasmid in the presence of a *Notch4* expression vector or the corresponding empty vector, with a full-length *Notch1* (Notch1) or *Notch1* intracellular domain (NIC1) expression vectors. Cells were activated with LPS for 24 h. The means ± SD of three independent experiments is shown. One-way ANOVA/Bonferroni’s post-tests were performed. ***p<0.001 compared to the corresponding LPS activated, Notch4 untransfected cells, ^###^p<0.001 compared to the corresponding LPS activated, non transfected with *Notch1* expression vectors cells.

NOTCH receptor signaling is finely controlled by the proteolytic action of three enzymes, furin, ADAM10 and the γ-secretase complex (13). As NOTCH4 seems to act as a negative regulator of CBF-1 activation by TLR4, we evaluated whether NOTCH4 specifically modulated the expression of the proteolytic enzymes involved in NOTCH receptor processing. We first analyzed the expression of these enzymes in macrophages with increased NOTCH4 levels and activated with LPS at different times. As shown in **Figure 6A**, high levels of NOTCH4 significantly lowered both furin mRNA and protein levels*. Notch4* overexpression reduced the proteolytic activity of ADAM10 by decreasing the formation of the 55 kDa catalytically active enzyme, compared to the control macrophages (**Figure 6A**). This reduction was expected, due to the lower level of expression of furin, one of the proteases that activate ADAM10 by cleaving the prodominium of the ADAM10 inhibitor (31).

We next evaluated whether the lower levels of furin observed in the case of NOTCH4 overexpression could affect the processing of the other NOTCH receptors. The quantity of the 120 kDa NOTCH1 transmembrane protein was reduced in macrophages with elevated *Notch4* expression activated with LPS at different times (**Figure 6B**). *Notch1* mRNA expression was also lower in overexpressing NOTCH4 macrophages than in controls (**Figure 6B**, lower panel). This result was expected, as NOTCH1 induces its own expression (32). These data suggest that NOTCH4 can inhibit the signaling activity of the other three NOTCH receptors, through the regulation of furin expression and ADAM10 activation. To prove this, we compared the effect of overexpressing NOTCH4 on the transactivation capacity of a CBF-1-dependent reporter gene activated by two different versions of NOTCH1, the full NOTCH1 receptor (Notch1) and the NOTCH1 intracellular domain (NIC1). Raw 264.7 cells were transfected with each of the two NOTCH1 constructs and activated with LPS for 24 hours. As **Figure 6C** shows, forced expression of both versions of the NOTCH1 receptor increased the CBF-1 reporter activity, both at the basal level and after activation with LPS. In these conditions, NOTCH4 overexpression decreased the luciferase activity induced by both NOTCH1 receptor forms, demonstrating that the inhibition that NOTCH4 exerts on NOTCH1 activation is not occurring at the level of NOTCH1 proteolytic processing. However, we cannot completely discard a partial effect of NOTCH4 on NOTCH1 processing, as N1C1 increases *Notch1* expression. New experiments are necessary to identify the mechanism by which NOTCH4 interferes with the transactivation capacity of the other NOTCH receptors.

### NOTCH4 Favors a HES1-Dependent STAT3 Transcriptional Activation

**Figure 7:**
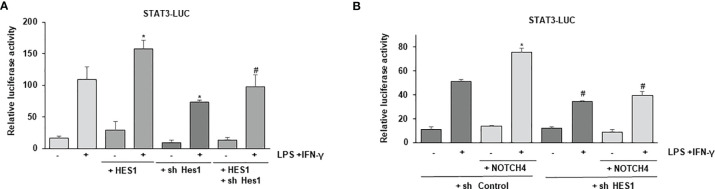
Effect of HES1 expression on the activation of STAT3 induced by NOTCH4 in macrophages treated with LPS and IFN-γ. **(A)** Analysis of luciferase activity in Raw 264.7 cells transiently transfected with a STAT-luciferase reporter in the presence of STAT3 (STAT3-LUC) and a *Hes1* expression vector or the corresponding empty vector, or with specific shRNA for *Hes1* or its control. One day after transfection, cells were stimulated with LPS (100 ng/ml) and IFN-γ (10 U/ml) for 24 h before analysis. The mean ± SD of three independent experiments is shown. One-way ANOVA/Bonferroni’s post-tests were performed. *p<0.05 compared to the control transfected cells activated with LPS and IFN-γ. ^#^p<0.05 compared to the cells transfected with *Hes1* and activated. **(B)** Analysis of luciferase activity in Raw 264.7 cells transiently transfected with a STAT-luciferase reporter in the presence of STAT3 (STAT3-LUC) and a *Notch4* expression vector or the corresponding control empty vector and with specific shRNA for *Hes1* or control shRNA. Cells were activated as previously described. The mean ± SD of three independent experiments is shown. One-way ANOVA/Bonferroni’s post-tests were performed. *p<0.05 compared to the control transfected cells activated with LPS and IFN-γ. ^#^p<0.05 compared to the activated cells transfected with control shRNA.

Previous reports have shown that HES1 can interact with STAT3, favoring its phosphorylation (33, 34). We speculated that the elevated levels of HES1 observed in the presence of NOTCH4 could explain the preferential phosphorylation and activation of STAT3 over STAT1 after IFN-γ treatment. In these conditions, the STAT1-dependent transcription program that characterizes IFN-γ signaling would be inhibited. To evaluate that, we first analyzed whether HES1 could control STAT3 activation in macrophages. As **Figure 7A** shows, HES1 acted as a positive STAT3 transcriptional regulator in macrophages, and this effect was diminished by treatment with a specific *Hes1* shRNA. We next evaluated whether HES1 could be responsible for the NOTCH4-dependent cross-regulation between STAT1 and STAT3 activities after LPS and IFN-γ signaling. As **Figure 7B** shows, NOTCH4 was unable to increase the STAT3 transcriptional activity in the presence of a specific *Hes1* shRNA, suggesting that the increase in STAT3 phosphorylation in the presence of NOTCH4 is mediated, at least in part, by HES1. On the contrary, the inhibitory effect exerted by NOTCH4 on STAT1 activity does not appear to be mediated by HES1 (**Supplementary Figure 7**).

### NOTCH4 Inhibits NF-κB Signaling in Activated Macrophages

**Figure 8:**
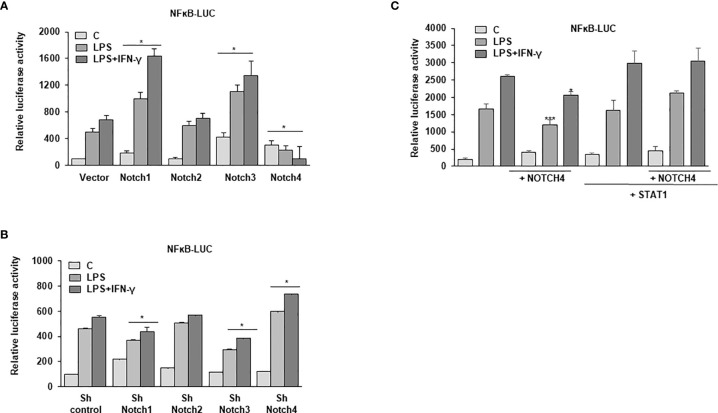
Analysis of the effect of NOTCH4 expression on NF-κB activation in LPS and IFN-γ activated macrophages. **(A)** Analysis of luciferase activity in Raw 264.7 cells transiently transfected with an NF-κB luciferase reporter gene (NF-κB-luc) and specific expression vectors for *Notch* receptor genes or their corresponding empty vectors, or **(B)** specific shRNAs for the different *Notch* receptor genes or control shRNA. One day after transfection, cells were stimulated with LPS (100 ng/ml) or LPS and IFN-γ (10 U/ml) for 24 h before analysis. The means ± SD of three independent experiments are shown. One-way ANOVA/Bonferroni’s post-tests were performed. *p<0.05 compared to the corresponding control transfected cells (vector or sh control). **(C)** Analysis of luciferase activity in Raw 264.7 cells transiently transfected with an NF-κB luciferase reporter gene (NF-κB-luc) and specific expression vectors for Notch4 receptor or STAT1 genes or their corresponding empty vectors. One day after transfection, cells were stimulated with LPS (100 ng/ml) or LPS and IFN-γ (10 U/ml) for 24 h before analysis. The means ± SD of three independent experiments are shown. One-way ANOVA/Bonferroni’s post-tests were performed. *p<0.05 and ***p<0.001, compared to the corresponding activated (LPS or LPS and IFN-γ) Notch4 untransfected cells.

IFN-γ plays a critical role in polarizing macrophages towards an M1 phenotype by enhancing the expression of pro-inflammatory cytokines induced by TLR activation (8, 35). Considering that one of the mechanisms by which IFN-γ favors the M1 phenotype is through enhancement of NF-κB signaling after TLRs activation (4), we decided to analyze the extent of activation of this transcription factor in NOTCH4-overexpressing cells in comparation with cells overexpressing other NOTCH receptors. We used an NF-κB-reporter gene in macrophages activated by LPS and IFN-γ and observed that, as previously described (9, 10), NOTCH1 and NOTCH3 increased NF-κB-reporter activity, whereas NOTCH2 had no significant effect. However, overexpression of NOTCH4 caused an inhibition of the NF-κB-reporter activity (**Figure 8A**). In agreement with these results, when the expression of NOTCH1 and NOTCH3 was diminished, a decrease in NF-κB-reporter activity was detected, as previously reported (9), whereas the decrease in NOTCH4 expression increased its activity in Raw 264.7 cells activated with LPS or with LPS and IFN-γ (**Figure 8B**). These results show that NOTCH4 activity exerts a negative regulation also on NF-κB-dependent transcription.

As one of the mechanisms by which IFN-γ increases NF-κB activity is by augmenting the expression of different components of the TLR pathway through STAT1 activation (36), we evaluated whether the lower STAT1 activation observed in the presence of high NOTCH4 levels could be responsible for the lower transcriptional activity of NF-κB. We analyzed the activity of an NF-κB reporter gene in Raw 264.7 cells transfected with *Notch4* and *Stat1* expression vectors and activated by LPS and IFN-γ. As **Figure 8C** shows, Raw 264.7 cells with forced expression of *Notch4* presented lower NF-κB activity, as described above. However, in the presence of high STAT1 levels, the inhibition of NF-κB activity by NOTCH4 was not observed. Thus, we conclude that NOTCH4 inhibits NF-κB at least in part through the inhibition of STAT1 activity.

## Discussion

Macrophages control the production of proinflammatory cytokines and effector mechanisms by numerous inhibitory processes aimed at preventing excessive toxicity and tissue damage. We have observed that NOTCH4 expression is increased in macrophages activated with pro-inflammatory stimuli, and we have investigated the role that NOTCH4 plays in this process. Our results show that this receptor acts as a negative regulator of macrophage proinflammatory activation, as it diminishes the expression of proinflammatory cytokines, including IL-6 and IL-12, and costimulatory molecules, such as CD80 and CD86. This effect was mediated through the inhibition of key transcription factors involved in this response, such as STAT1 and NF-κB. Moreover, NOTCH4 inhibits canonical CBF-1-dependent NOTCH transcription in activated macrophages, and this process could also alter the expression of proinflammatory genes.

**Figure 9:**
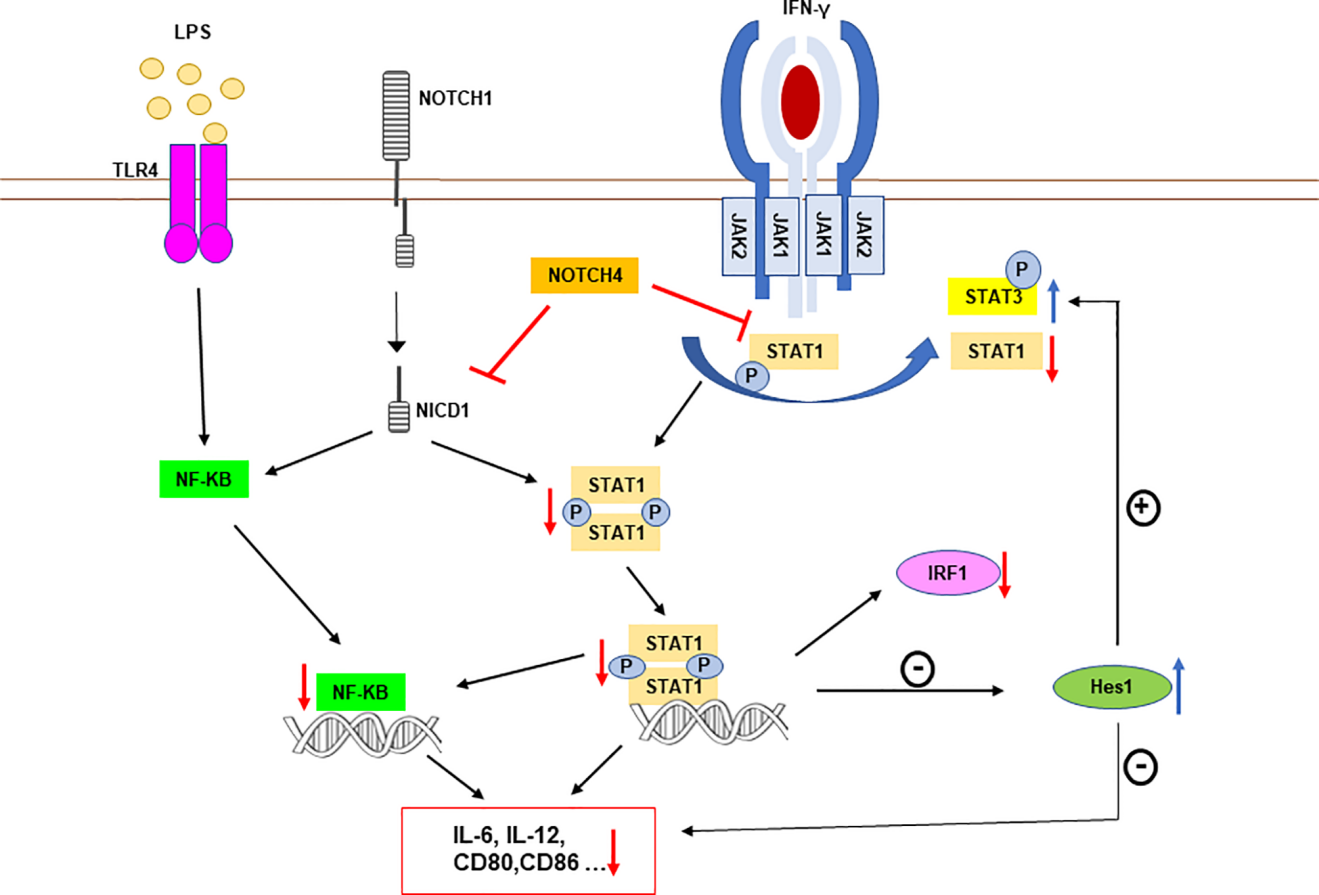
Schematic representation of NOTCH4 receptor interference with TLR4 and IFNγ receptor signaling in proinflammatory activated macrophages. NOTCH4 diminishes STAT1 phosphorylation after IFN-γ receptor activation. This lower STAT1 activation decreases the expression of STAT1-dependent genes, including IRF1, but increases the expression of HES1, which is normally repressed by STAT1 (39). HES1 exerts an anti-inflammatory action by inhibiting the expression of genes such as those for IL-6 or IL-12 (8). Moreover, enhanced levels of HES1 could facilitate STAT3 phosphorylation and activation, increasing the expression of anti-inflammatory genes, such as that for IL-10 (7). NOTCH4 also inhibits NF-κB-dependent transcription. This effect is mediated, at least in part, by the lower activation of STAT1, that normally cooperates with NF-κB increasing its transcriptional activity in some pro-inflammatory gene promoters, such as those of IL-6 or IL-12 (36). Because of the lower STAT1 and NF-κB activation mediated by NOTCH4, and of the elevated levels of the repressor HES1, the expression of STAT1 and NF-κB target genes, such as IL-6, IL-12 or CD80 among others, is diminished. The blue arrows mark increased phosphorylation or expression induced by NOTCH4, whereas red arrows indicate repression by NOTCH4.

IFN-γ is a pluripotent cytokine whose main biological effects are mediated by the activation of the STAT1 transcription factor (37, 38), although it also causes a lower and transient activation of STAT3. Our results show that NOTCH4 decreases the macrophage response to IFN-γ through inhibition of STAT1 phosphorylation at Tyr701, which would limit its dimerization, entry into the nucleus and binding to GAS sequences present on target genes (**Figure 9**). On the contrary, NOTCH4 increases STAT3 phosphorylation at Tyr705. STAT1 and STAT3 exert opposite effects on the immune response: signaling through STAT1 promotes inflammation by enhancing the expression of genes encoding proteins of the importance of iNOS, IL-1*2*, IL-6, CCL2, MCP1, CXCL10 or MHC-I (40, 41), whereas STAT3 acts as a negative regulator of signaling cascades triggered by LPS, IFN-γ and other cytokines, by inhibiting the expression of inflammatory cytokines through the expression of genes such as *SOCS3* or that encoding for IL-10 (7). Our results show that NOTCH4 reprograms the macrophage response to IFN-γ, favoring STAT3 *versus* STAT1 phosphorylation without affecting their expression.

Previous studies have shown that STAT1 and STAT3 can regulate each other. In *STAT1* ^-/-^ embryonic fibroblasts or macrophages, IFN-γ produces a stronger and longer activation of STAT3 and its target genes than in control cells, suggesting a negative regulation of STAT3 by STAT1 (28, 42). A recent study in squamous cells of an esophagus carcinoma model shows that these cells present increased STAT3 phosphorylation and signaling due to a constitutive activation of ERK, which promotes the degradation of STAT1 *via* the proteasome (43). In contrast with these results, we observed a switch in STAT1/STAT3 phosphorylation and activation without changes in the expression of these proteins, pointing to the existence of two different regulatory mechanisms for these processes.

STAT1 and STAT3 are phosphorylated by the Janus kinases (JAK1 and JAK2) associated with the IFN-γ receptor, and both proteins seem to compete for the same binding site (28). Other kinases, such as Src kinases, can be activated by IFN-γ though phosphorylation mediated by JAK1 and JAK2, further contributing to the activation of STAT3, but not STAT1 (28, 44). Here, we show that JAK2 is required for the phosphorylation of STAT3, although we still cannot identify JAK2 as a direct target of NOTCH4.

We have observed that HES1 can mediate, in part, the enhancement of STAT3 activation induced by NOTCH4. These results are in agreement with previous work showing that HES1 facilitates the formation of a complex between JAK2 and STAT3 through binding to these proteins, thus promoting STAT3 phosphorylation and activation after growth factor stimulation of glial cells (34). Similarly, a recent study in colon epithelial cells shows that HES1 increases STAT3 activity and thus favors tumorigenesis (45). Although STAT1 is preferentially activated in response to IFN-γ in macrophages, NOTCH4-dependent rise of HES1 expression could favor the interaction of STAT3 with JAK2, favoring this activation rather than that of STAT1 with JAK2. Nevertheless, NOTCH4 could also directly or indirectly interact with STAT1, making it less accessible to IFN-γ receptor-associated kinases. This could also lower STAT1 activation, leading to increased HES1 levels, since it has been described that STAT1 acts as a negative regulator for *Hes1* expression (39). In this way, NOTCH4 would then enhance STAT3 activation on a HES1-dependent manner as well, which would add to the direct effect of NOTCH4 on STAT1 (**Figure 9**).

Our results also show that, during macrophage activation, NOTCH4 inhibits the canonical RBP-Jκ/CBF-1 NOTCH signaling pathway. Indeed, NOTCH4 overexpression is unable to induce the activation of the canonical NOTCH signaling, as all other NOTCH receptors do, even when macrophages are activated through TLR. The inability of NOTCH4 to activate CBF-1 has been previously described (46). In addition, NOTCH4 has been shown to interact with the entire NOTCH1 receptor, causing inhibition of the NOTCH1 receptor signaling, probably by limiting its proteolytic activation process (14). In macrophages, we also observed that NOTCH4 inhibits the overall NOTCH signaling pathway, partly through inhibition of NOTCH1, but unlike that observed in endothelial cells (14), in macrophages, NOTCH4 can also inhibit NICD1 signaling. This seems to indicate that this process is independent of NOTCH1 proteolytic processing. Nevertheless, we observed a lower induction of furin expression in macrophages with elevated NOTCH4 expression levels and, consequently, a lower activation of ADAM10. Both effects, that dependent on and that independent on receptor processing, could be responsible for the inhibition of global NOTCH signaling by NOTCH4. We and others have observed that induction of furin after TLR activation depends on NOTCH1 activity (9, 47). The inhibition of NOTCH1 signaling by NOTCH4 could also be related to the lower activation of STAT1 and NF-κB observed in macrophages with high NOTCH4 expression (**Figure 9**), as NOTCH1 has been shown to increase the activity of these transcription factors (9, 10, 48).

Another relevant result of this work is the unveiling of NOTCH4 ability to reverse the inhibitory effect that IFN-γ exerts on NOTCH signaling. A previous comparative expression study performed on human macrophages activated by TLRs, in the presence or in the absence of IFN-γ, showed that *Hes1* and *Hey1*, two NOTCH-target genes, were induced by TLR triggering and suppressed by IFN-γ (8). Our group has also observed these inhibitory effects of IFN-γ on NOTCH signaling in murine macrophages (10). NOTCH4 could modulate the macrophage inflammatory profile through this mechanism, i.e. by reversing the inhibitory effect of IFN-γ on the CBF-1 activity over its target genes, *Hes1* and *Hey1*, which have been shown to attenuate the expression of cytokines, such as IL-6 and IL-12 (8). Thus NOTCH4, through the inhibition of the IFN-γ signal, would favor global NOTCH signaling by promoting the feedback inhibitor loop carried out by HES1 and HEY1, which decrease the production of cytokines. Indeed, as it was described above, NOTCH4 reprograms the macrophage response to IFN-γ, favoring STAT3 *versus* STAT1 phosphorylation, partially though HES1. Our results provide new insights into the mechanisms by which NOTCH, TLR and IFN-γ signals are integrated to modulate specific effector functions in macrophages. However, understanding the exact molecular mechanism by which NOTCH4 regulates the IFN-γ signal requires further research.

Some studies have previously related NOTCH4 with the inflammatory response, like those describing that *Notch4* ^-/-^ mice show increase sensitivity to pulmonary inflammation after ozone treatment (17) or, more recently, another study reporting that *Notch4^-/-^* mice were more resistant than control mice to infection caused by *Mycobacterium tuberculosis* (15). Our results agree with both studies, as we also observed lower expression and production of the proinflammatory cytokines IL-6 and IL-12 when NOTCH4 levels were elevated, whereas, on the contrary, higher expression of these cytokines was observed when NOTCH4 levels were diminished. Certainly, in addition to the inhibitory effect that NOTCH4 exerts on IFN-γ signaling, by diminishing STAT1 activation, we have observed that NOTCH4 decreases NF-κB activation. NF-κB is an essential transcription factor for the expression of multiple pro-inflammatory genes, such as IL-12 or IL-6, or coestimulatory molecules, such as CD80 and CD86 (49, 50). In that sense, it has been described that, after IFN-γ signaling, STAT1 could access canonical NF-κB-target genes, such as IL-6, that do not contain GAS elements, favoring their transcription. This process is mediated by the recruitment of histone acetyltransferases and other chromatin-remodeling enzymes (36). In agreement with this, we have observed that inhibition of NF-κB-dependent transcription by NOTCH4 is reduced in the presence of high levels of STAT1, so that the inhibitory effect of NOTCH4 on NF-κB could be due to reduced STAT1 activation (**Figure 9**). Nevertheless, a previous work has described that NOTCH4 interacts with TAK1 and Traf6 in macrophages, thus limiting the activation of TAK1 (15), which is one of the main regulators of NF-κB. It has also been described that NOTCH4 deficient mice have increased Traf6 expression, which also favors the activation of NF-κB (17). All this evidence provides two alternative non-exclusive mechanisms by which NOTCH4 negatively regulates the inflammatory response in macrophages.

All these results reveal an important role of NOTCH4 in the development and resolution of immune responses by controlling the immunostimulatory effects of macrophages on NK and Th1 cell activation and differentiation. These effects are exerted through the control of the production of cytokines, such as IL-12, or costimulatory molecules, such as CD80 and CD86, which play an essential role in the control of viral and bacterial infections. Moreover, NOTCH4 favors STAT3 activation. Hyperactivation of STAT3 in antigen-presenting cells has been associated with the secretion of immunosuppressive factors, such as IL-10 and TGF-β, that contribute to the establishment of a tolerogenic microenvironment (51). Thus, NOTCH4 seems to act to avoid an excessive and potentially harmful immune response. On the other hand, an excessive NOTCH4 activity could compromise the adequate defense of the organism against infections.

Different studies have related certain NOTCH4 polymorphisms with the development of inflammatory processes associated with autoimmune diseases, including rheumatoid arthritis or alopecia areata (52). NOTCH4 expression is also induced by glucocorticoids, an important family of anti-inflammatory drugs, although its role on glucocorticoid anti-inflammatory activity has not yet been established (53). However, other studies show that a *Notch4* missense mutation is associated with an increased susceptibility to tuberculosis in the Chinese population (54). NOTCH4 seems to be also implicated in allergic airway inflammation induced by alveolar macrophages exposed to ultrafine particles (55). Although further work is necessary to refine our understanding about the role that NOTCH4 plays in inflammation, our results clearly show it interferes with key transcription factors implicated in macrophage proinflammatory activation. Our results, therefore, provide novel avenues for therapeutic intervention in these processes.

## Data Availability Statement

The raw data supporting the conclusions of this article will be made available by the authors, without undue reservation.

## Ethics Statement

The studies involving human participants were reviewed and approved by Comite de Etica del Complejo Hospitalario Universitario de Albacete. The patients/participants provided their written informed consent to participate in this study. The animal study was reviewed and approved by Comité de Ética de Experimentación Animal de la Universidad de Castilla la Mancha.

## Author Contributions

MD-G and EM conceived and designed the research. SL-L, EM, MR, JG-R, NH, FR-M, VB, and MN performed the experiments MD-G, JG-R, and JL analyzed de data and prepared the manuscript. All authors contributed to the article and approved the submitted version.

## Funding

This work was supported by a grant from Consejería de Educación, Cultura y Deporte, Junta de Comunidades de Castilla-La Mancha, Spain (grant SBPLY/17/180501/000301), the Instituto Carlos III, Ministerio de Economía y Competitividad, Spain (grant PI15/00991), and the Ministerio de Ciencia e Innovación, Spain (grant PID2019-109421RB-100).

## Conflict of Interest

The authors declare that the research was conducted in the absence of any commercial or financial relationships that could be construed as a potential conflict of interest.

## Publisher’s Note

All claims expressed in this article are solely those of the authors and do not necessarily represent those of their affiliated organizations, or those of the publisher, the editors and the reviewers. Any product that may be evaluated in this article, or claim that may be made by its manufacturer, is not guaranteed or endorsed by the publisher.
