# Supplementary figures and images for: NOTCH4 Exhibits Anti-Inflammatory Activity in Activated Macrophages by Interfering With Interferon-γ and TLR4 Signaling
Source: Front Immunol. 2021 Dec 1;12:734966. doi: 10.3389/fimmu.2021.734966 (PMC8671160; doi:10.3389/fimmu.2021.734966)

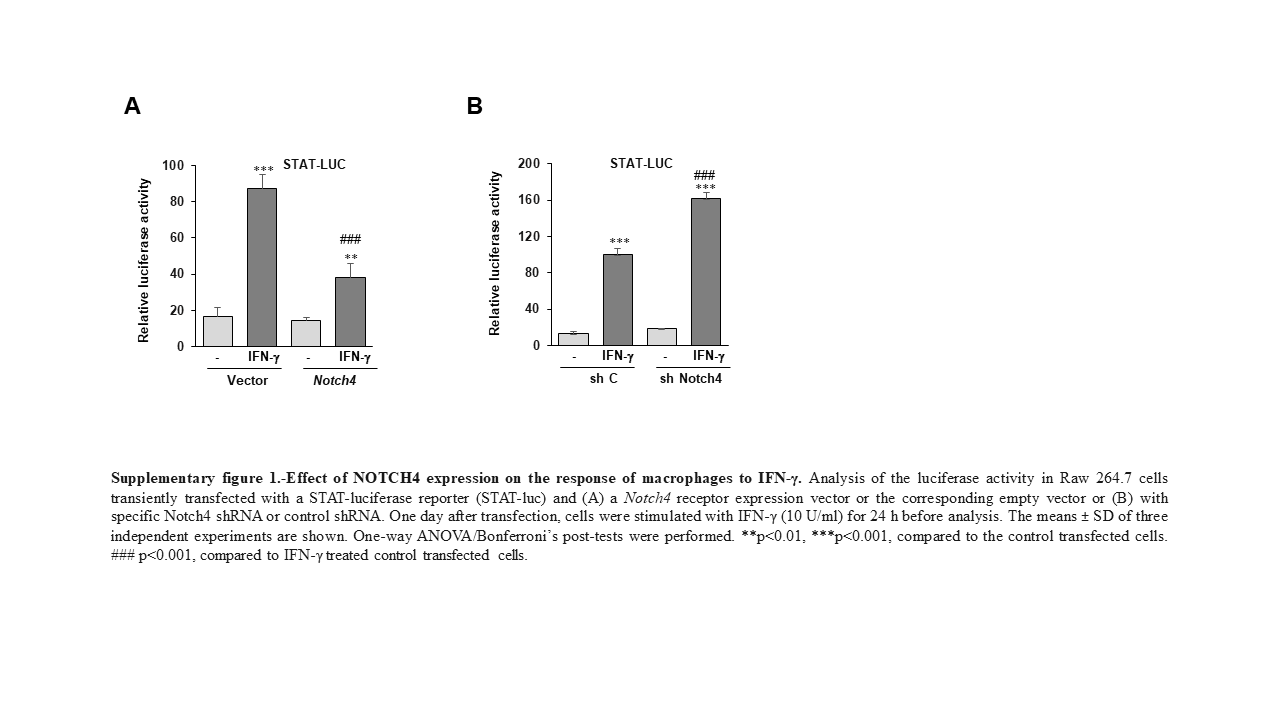

Supplement: Supplementary file 1 [file Image_1.tif]

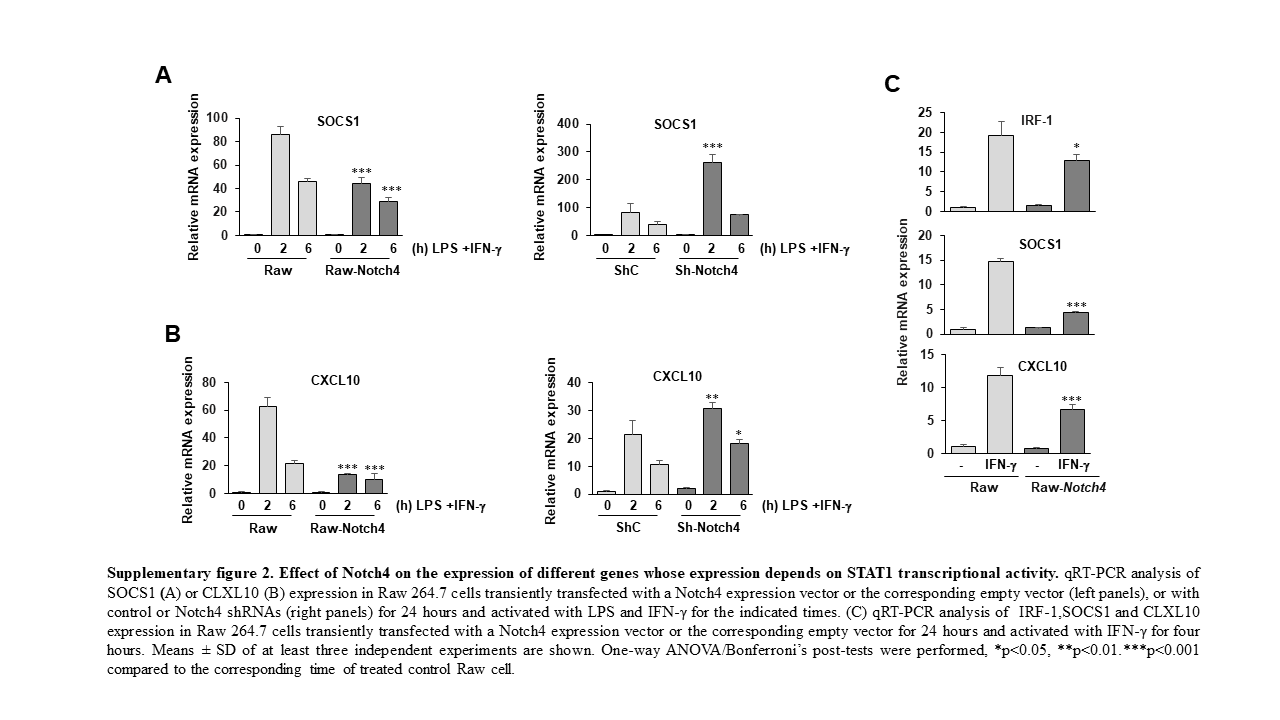

Supplement: Supplementary file 2 [file Image_2.tif]

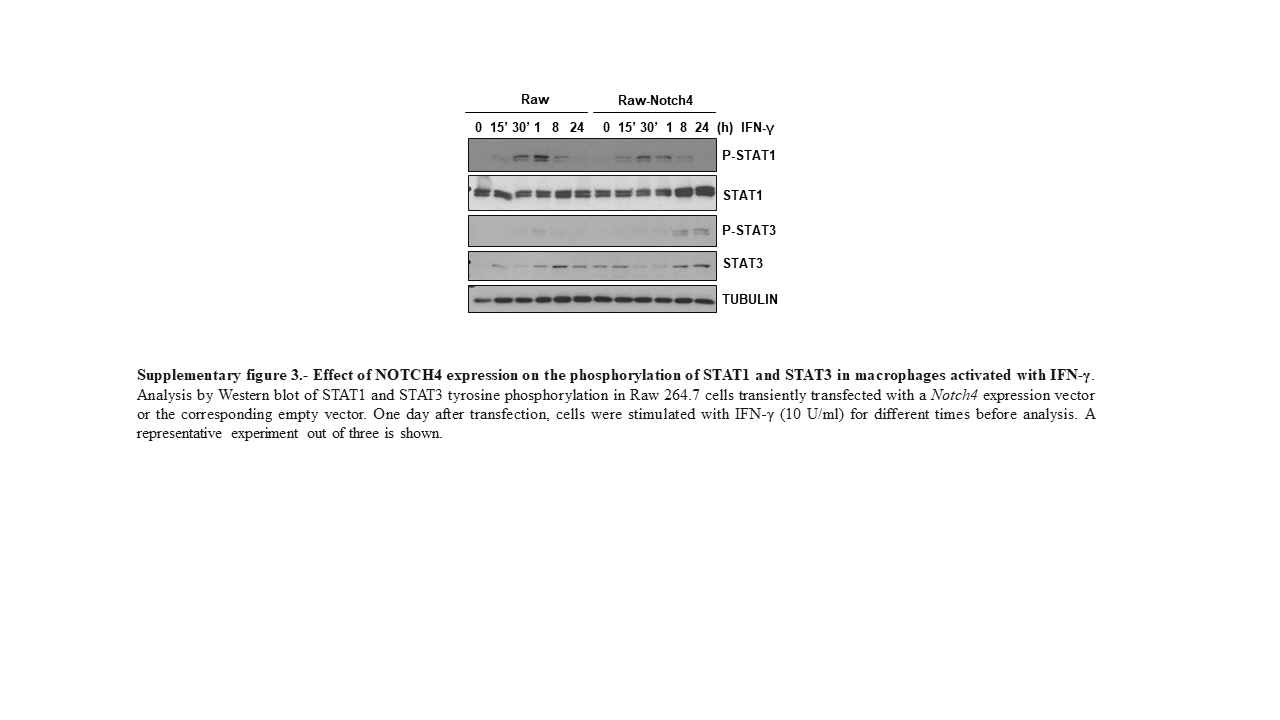

Supplement: Supplementary file 3 [file Image_3.tif]

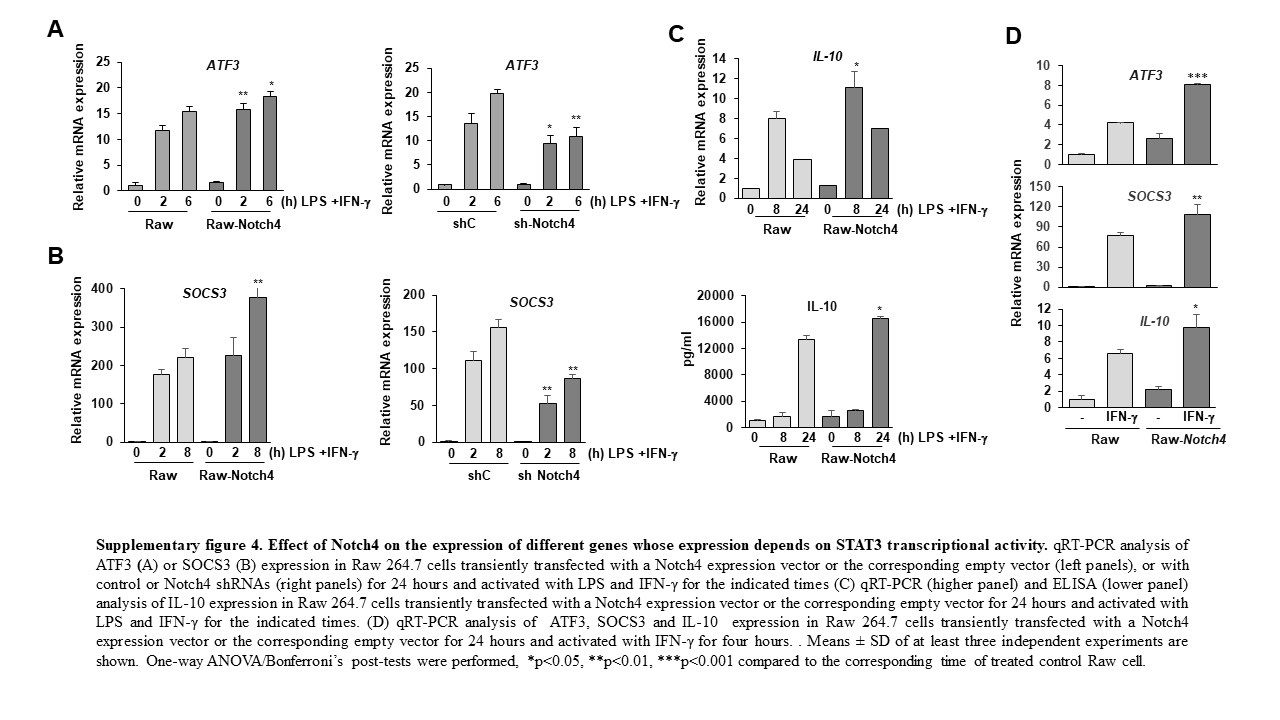

Supplement: Supplementary file 4 [file Image_4.tif]

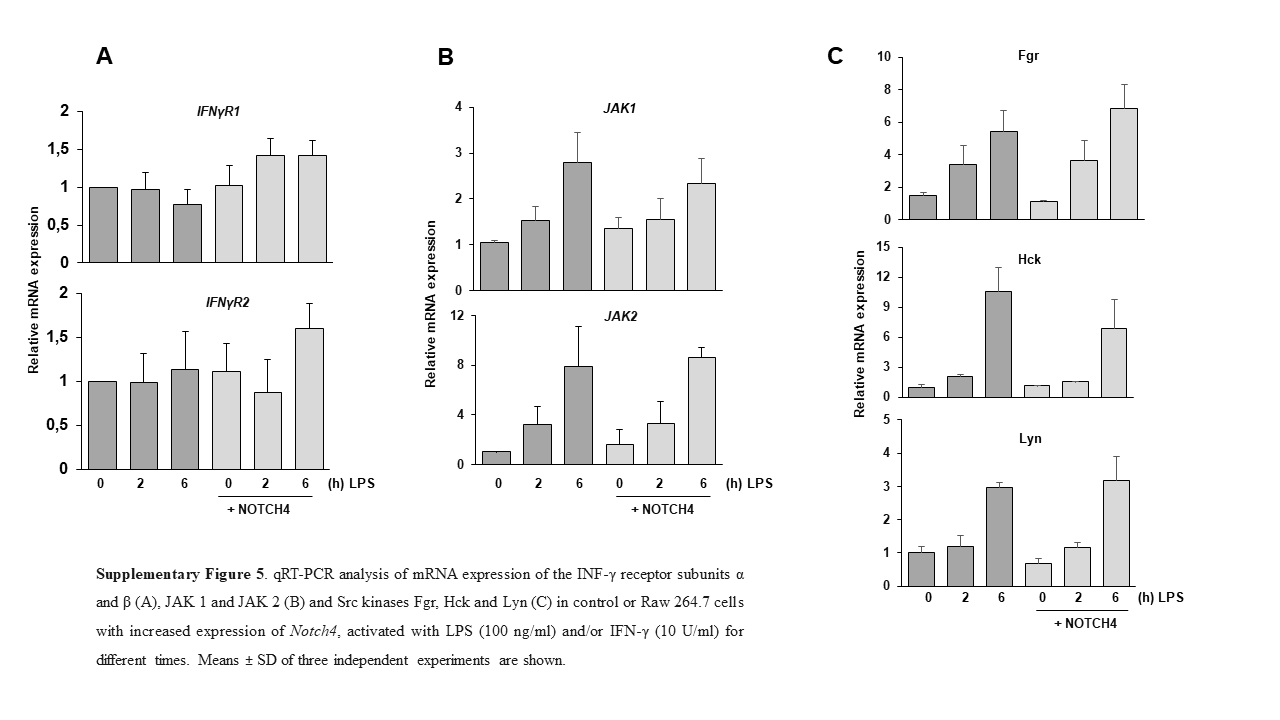

Supplement: Supplementary file 5 [file Image_5.tif]

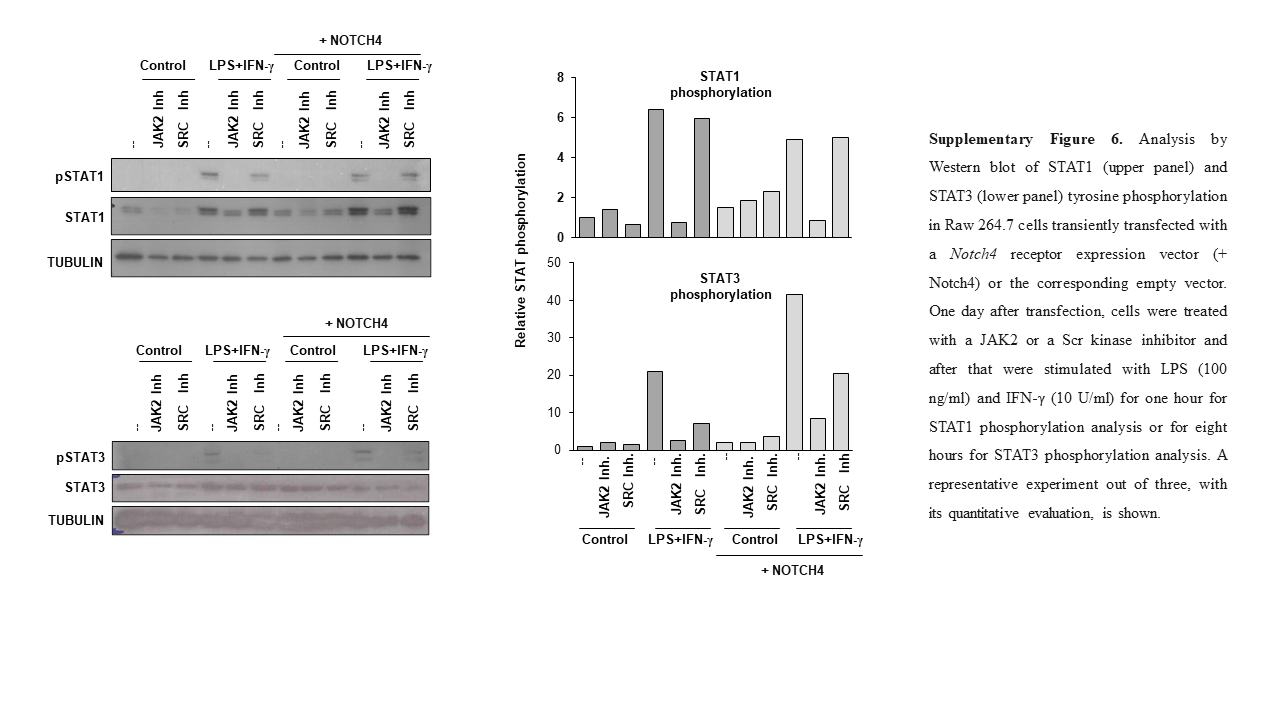

Supplement: Supplementary file 6 [file Image_6.tif]

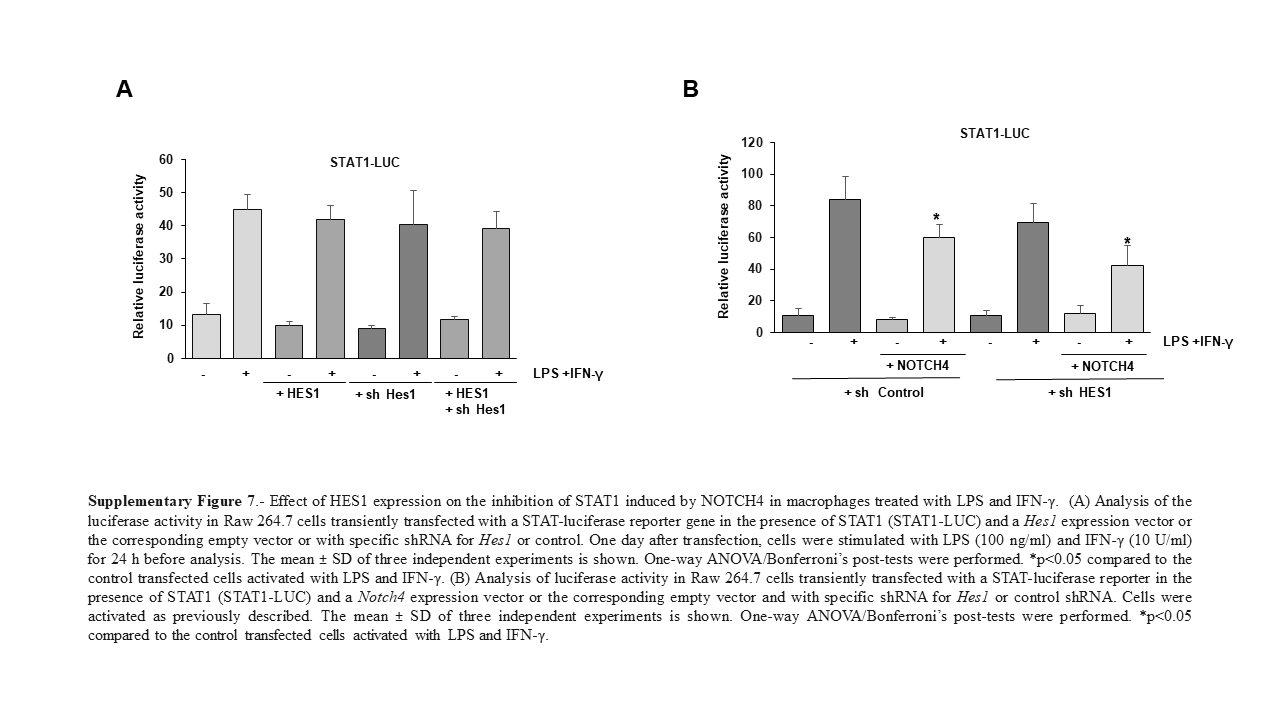

Supplement: Supplementary file 7 [file Image_7.tif]
